# The Effect of Robotic Assisted Gait Training With Lokomat® on Balance Control After Stroke: Systematic Review and Meta-Analysis

**DOI:** 10.3389/fneur.2021.661815

**Published:** 2021-07-06

**Authors:** Federica Baronchelli, Chiara Zucchella, Mariano Serrao, Domenico Intiso, Michelangelo Bartolo

**Affiliations:** ^1^Physiotherapy Center Kiné, Cremona, Italy; ^2^Neurology Unit, University Hospital of Verona, Verona, Italy; ^3^Department of Medico-Surgical Sciences and Biotechnologies, Sapienza University of Rome Polo Pontino, Latina, Italy; ^4^Unit of Neuro-Rehabilitation and Rehabilitation Medicine, Istituto di Ricovero e Cura a Carattere Scientifico (IRCCS) Casa Sollievo della Sofferenza, San Giovanni Rotondo, Italy; ^5^Neurorehabilitation Unit, Department of Rehabilitation, HABILITA Zingonia, Ciserano, Italy

**Keywords:** balance, Lokomat®, exoskeleton, robotics, stroke, neurorehabilitation, gait

## Abstract

**Introduction:** Disturbances of balance control are common after stroke, affecting the quality of gait and increasing the risk of falls. Because balance and gait disorders may persist also in the chronic stage, reducing individual independence and participation, they represent primary goals of neurorehabilitation programs. For this purpose, in recent years, numerous technological devices have been developed, among which one of the most widespread is the Lokomat®, an actuated exoskeleton that guide the patient's limbs, simulating a symmetrical bilateral gait. Preliminary evidence suggests that beyond gait parameters, robotic assisted gait training may also improve balance. Therefore, the aim of this systematic review was to summarize evidence about the effectiveness of Lokomat® in improving balance in stroke patients.

**Methods:** Randomized controlled trials published between January 1989 and August 2020, comparing Lokomat® training to conventional therapy for stroke patients, were retrieved from seven electronic databases. Balance, assessed by means of validated clinical scales, was considered as outcome measure. The Physiotherapy Evidence Database (PEDro) scale was used to evaluate the methodological quality of the studies. The study protocol was registered on PROSPERO (no. CRD42020197531).

**Results:** After the removal of the duplicates, according to the inclusion criteria, 13 studies were selected, involving 445 subacute or chronic stroke patients. Eleven papers contributed to three meta-analyses. Favorable results for recovery of balance in stroke survivors treated with Lokomat® were shown using Timed Up and Go (pooled mean difference = −3.40, 95% CI −4.35 to −2.44; *p* < 0.00001) and Rivermead Mobility Index as outcome measures (pooled mean difference = 0.40, 95% CI 0.26–0.55; *p* < 0.00001). Inconclusive results were found when balance was measured by means of the Berg Balance Scale (pooled mean difference = 0.17, 95% CI −0.26 to 0.60; *p* = 0.44).

**Conclusions:** Overall, most studies have shown beneficial effects of Lokomat® on balance recovery for stroke survivors, at least comparable to conventional physical therapy. However, due to the limited number of studies and their high heterogeneity, further research is needed to draw more solid and definitive conclusions.

## Introduction

Balance control is described as a complex motor skill that depends on interactions between multiple sensorimotor processes and environmental and functional contexts that in stroke survivors can be affected individually or in combination, producing balance disorders of different severity (1). Balance is an essential component of the quality of walking (2–4), and in stroke survivors, balance impairment may determine abnormal patterns of gait characterized by decreased walking speed and stride length and spatial and temporal asymmetry (5–9) that in turn increase the risk of falling (10–14). Because balance and gait disorders may persist also in the chronic stage (15), they represent one of the main limiting factors for individual independence and participation in social activities, reducing patients' quality of life (16–18).

As it is well-established that balance control strategies can become more efficient with training and practice, improving balance and gait represents a main goal of neurorehabilitation programs to restore effective and safe mobility (19–22). Although the development of robotic devices that mechanically guide patient's limbs through the gait cycle is a relatively novel approach, in recent years, many devices were developed and have become commercially available. Among these robot machines, the Lokomat® (Hocoma AG, Volketswil, Switzerland) is an actuated exoskeleton that simulates a symmetrical bilateral gait while the individual walks more or less actively on a treadmill; this device helps the patient reproduce the different phases of the physiological gait cycle, fragmenting the joints, symmetrically balancing the load, and transmitting proprioceptive afferents. Studies have shown that the rhythmic and repetitive step pattern provided by the robotic guidance combined with the active load of the limbs and kinematic coherence promotes the plasticity of the gait pattern generator, facilitating motor schemes and neural plasticity at the spinal cord and supraspinal level (23).

Most of the previous researches evaluating the effectiveness of Lokomat® focused on the kinematic parameters of gait in patients affected by stroke (24) or spinal cord injury (25), while only a few studies focused on balance as outcome measure.

Notwithstanding the small number of studies, two systematic reviews have been published that provided preliminary evidence about the effect of robot-assisted gait training (RAGT) and reported that rehabilitative robotic strategy can improve balance for stroke patients similarly to conventional gait rehabilitation (26, 27). However, these systematic reviews and meta-analyses considered variable kinds of robotic devices including both static exoskeletons and end-effector machine that may involve different neural mechanisms in the recovery process. Although RAGT/Lokomat® was also included, no data and remark could be obtained about the effect of this robotic instrument on balance.

Moreover, it should be noted that, contrary to what happens in the clinical pharmacological field, as already discussed in the review by Iosa et al. (28), the introduction of robotic technologies in rehabilitation does not provide for preclinical efficacy studies, thus allowing the marketing of devices for which the main purpose of use is generally known, but not the effects on aspects functionally related to the main purpose.

Therefore, with the aim to increase knowledge about the specific effect of Lokomat® on improving balance control in stroke survivors, as well as to favor a more appropriate use of this robot, we systematically synthesize evidence and discuss data on this issue.

## Methods

This systematic review was reported according to the Preferred Reporting Items for Systematic reviews and Meta-Analysis statement (29, 30). All the analyses were performed on previous published papers, and the study was notified to the local ethics committee, according to current legislation. The study was performed in accordance with the Declaration of Helsinki, and the overview protocol was registered on PROSPERO (no. CRD42020197531).

### Eligibility Criteria

Objectives were defined according to the PICO model (Population, Intervention, Comparison, Outcome type). The population of interest was adults post-stroke (both ischemic and hemorrhagic). Interventions considered were rehabilitation treatment performed by means of the Lokomat® robotic device, with no restrictions on the setting (inpatients and outpatients). Comparison groups included other rehabilitation methods (conventional physiotherapy and/or treadmill) (see **Table 2** for a description of control therapies). Outcome considered was balance control, assessed by means of standardized clinical scales (44). Only studies written in English were considered.

Exclusion criteria were study design other than randomized controlled trial (RCT), studies involving subjects suffering from neurological diseases other than stroke, comparison of rehabilitation treatment performed by means of the Lokomat® with other unconventional therapies (e.g., electrical stimulation), studies with full text not available, non-peer-reviewed articles, congress abstracts/posters.

### Exoskeleton Lokomat®

The Lokomat® (Hocoma AG, Volketswil, Switzerland) is a robotic bilateral orthosis used in neurological rehabilitation to automate locomotor function. Briefly, it is composed of a body weight support system used in combination with a treadmill that replicates the lower extremity biomechanics of walking overground, sometimes associated with an augmented reality system. Lokomat® can be classified as an “exoskeletal robot.” In this kind of robot, knee and hip are driven by linear electrical motors that guide the external orthosis applied to the body, while a foot lift induces passive dorsiflexion of the ankle during the swing phase (45). This facilitates a bilateral symmetrical gait, as the individual actively tries to advance each limb while walking on the treadmill. The preprogrammed gait model corresponds to the normal kinematics of gait that includes the synchronization of the gait cycle, the coordination between the limbs and joints, and the appropriate load of the limbs (46, 47).

### Outcome Measures

For this meta-analysis, according to the International Classification of Functioning, Disability and Health (ICF), we considered balance as a level of activity reflecting functional abilities and postural control as a function of body structure reflecting both orientation and stabilization of the body (48). Therefore, in the included studies, the following standardized clinical scales were considered outcome measures:

- Berg Balance Scale (BBS): the scale that evaluates the patient's ability to safely maintain balance during a series of predetermined static and dynamic functional tasks. It is a 14-item list with each item consisting of a 5-point ordinal scale ranging from 0 (minimum performance) to 4 (best performance) with a maximum total score of 56 (≤ 45: need for travel assistance; 41–44: low risk of falling; 21–40: high risk; 0–20: very high risk of falling). It does not include the assessment of gait (49).
- Timed Up and Go (TUG): the test that determines fall risk and measures the progress of balance, sit to stand, and walking. The scale measures the time to get up from a chair, walk 3 m, turn around, go back to the chair, and sit down again. Subjects with a score ≥14 s are considered at risk of falling; the higher the score, the greater the risk (50).
- Rivermead Mobility Index (RMI): the 15-item questionnaire that investigates mobility in activity of daily living such as turning in bed, transfers, walking, going upstairs, running. The examiner is required to make one observation (standing unsupported >10 s), and all items are rated in a yes/no format with positive responses scoring a 1 for a maximal RMI score of 15. The higher the score, the better the performance (51).
- Modified Emory Functional Ambulation profile (mEFAP): the test that measures the time to ambulate through five common environmental terrains (floor, carpet, “up and go,” obstacles, and stairs) with or without an assistive device or manual assistance. The five timed subscores are added to derive a total score. The lower the score, the better the performance (52).
- Mobility Milestones (MM): the outcome measure that evaluates the time to achieve five mobility milestones: 1-min sitting balance, 10-s standing balance, a 10-step walk, a 10-m walk, a 6-min walk. The total score is obtained by adding up the time of each task. The higher the score, the longer the patient's recovery time (53).
- Postural Assessment Scale for Stroke (PASS): the 12-item performance-based scale used for assessing and monitoring postural control after stroke. The score of each item ranges from 0 to 3; therefore, the total score ranges from 0 to 36. The higher the score, the better the performance (54).
- Tinetti Performance-Oriented Mobility Assessment (POMA-B): the task-oriented test that measures an adult's balance abilities through an ordinal scale ranging from 0 (most impairment) to 2 (independence). The maximum score is 16. The lower the score, the greater the risk of falling (55).
- Short Physical Performance Battery (SPPB): the group of measures that combines the results of the gait speed, chair stand, and balance tests. The scores range from 0 (worst performance) to 12 (best performance) (56).
- Rivermead Motor Assessment (RMA): the evaluation that assesses functional mobility following stroke (e.g., gait, balance, transfers). It consists of three sections: gross function (RMA-gf), legs and trunk (RMA-lt), arm (RMA-a). Each item is scored either yes “1” or no “0.” The items in each section were ordered so that they were increasingly difficult for most patients, that is, a hierarchical scale. When a patient has failed one item, it is assumed that subsequent items will also be failed. For this reason, not all items need to be administered (57).

### Search Strategy

A search of relevant studies was conducted in MEDLINE/PubMed, Physiotherapy Evidence Database (PEDro), the Cochrane Central Register of Controlled Trials, CINAHL, EMBASE, Web of Science, and Scopus databases. We included reports from the international literature published between January 1989 and August 2020. Search terms “included stroke,” “cerebrovascular,” “cerebrovascular disease,” “ischemic stroke,” “hemorrhagic stroke,” “brain injury,” “chronic stroke,” “Lokomat,” “robotic device,” “exoskeleton,” “balance,” “postural balance,” “equilibrium,” “postural control,” “balance control,” “gait,” “walking,” “step,” “weight-bearing,” “locomotion,” “balance training,” “gait training,” and “walking training.” We searched for “randomized controlled trial” as MeSH terms, keywords, or subject headings. Related terms were combined using the Boolean “OR” and “AND.” In order to avoid bias, the search was repeated over several days, also changing the order of the keywords.

### Study Selection and Data Extraction

Two authors (FB, MB) independently screened titles and abstracts of the potentially relevant papers and removed the duplicates. Then, a careful check of the papers was performed in order to select only the studies that met the inclusion criteria. The reference lists of relevant papers were also inspected for additional studies potentially missed in the databases search. Any disagreement was solved by confrontation and consensus without involving a third author.

From each study included in the review, the same two authors (FB, MB) independently extracted the following data: title, authors, year of publication, journal of publication, participants (number, mean age, gender), study design, rehabilitative intervention details (frequency and duration of the sessions, Lokomat® parameters such as weight support and speed), outcome measures, results, follow-up.

### Evaluation of the Methodological Quality of the Studies

The methodological quality of the studies included in this review was measured through the use of the PEDro scale (58, 59). The PEDro scale includes 10 items (random allocation, concealed allocation, similarity at baseline, subject blinding, therapist blinding, assessor blinding, >85% follow-up for at least one key outcome, intention-to-treat analysis, between-group statistical comparison for at least one key outcome, and point and variability measures for at least one key outcome), each scored as present (1) or absent (0). Criteria that are not specified must be considered as not met. The criteria classified as achieved can be added to obtain a score between 0 (minimum) and 10 (maximum), which indicates the general methodological quality of the study (≤ 4 poor, 4–5 acceptable, 6–8 good, 9–10 excellent) (60).

### Data Analysis

A meta-analysis was performed to compare changes (pre- and post-intervention) between the study group (RAGT with Lokomat®) and the control group (conventional physiotherapy and/or treadmill). The studies were grouped according to the outcome measure, and for each meta-analysis, the standardized mean difference along with the 95% confidence interval (CI) was calculated. *p* < 0.05 was considered significance level. Heterogeneity was determined by the chi-square test and the I2 statistic. All statistical analyses were carried out by means of the Review Manager (RevMan) Software, Version 5.3 (The Cochrane Collaboration, The Nordic Cochrane Centre, London, UK).

## Results

**Figure 1:**
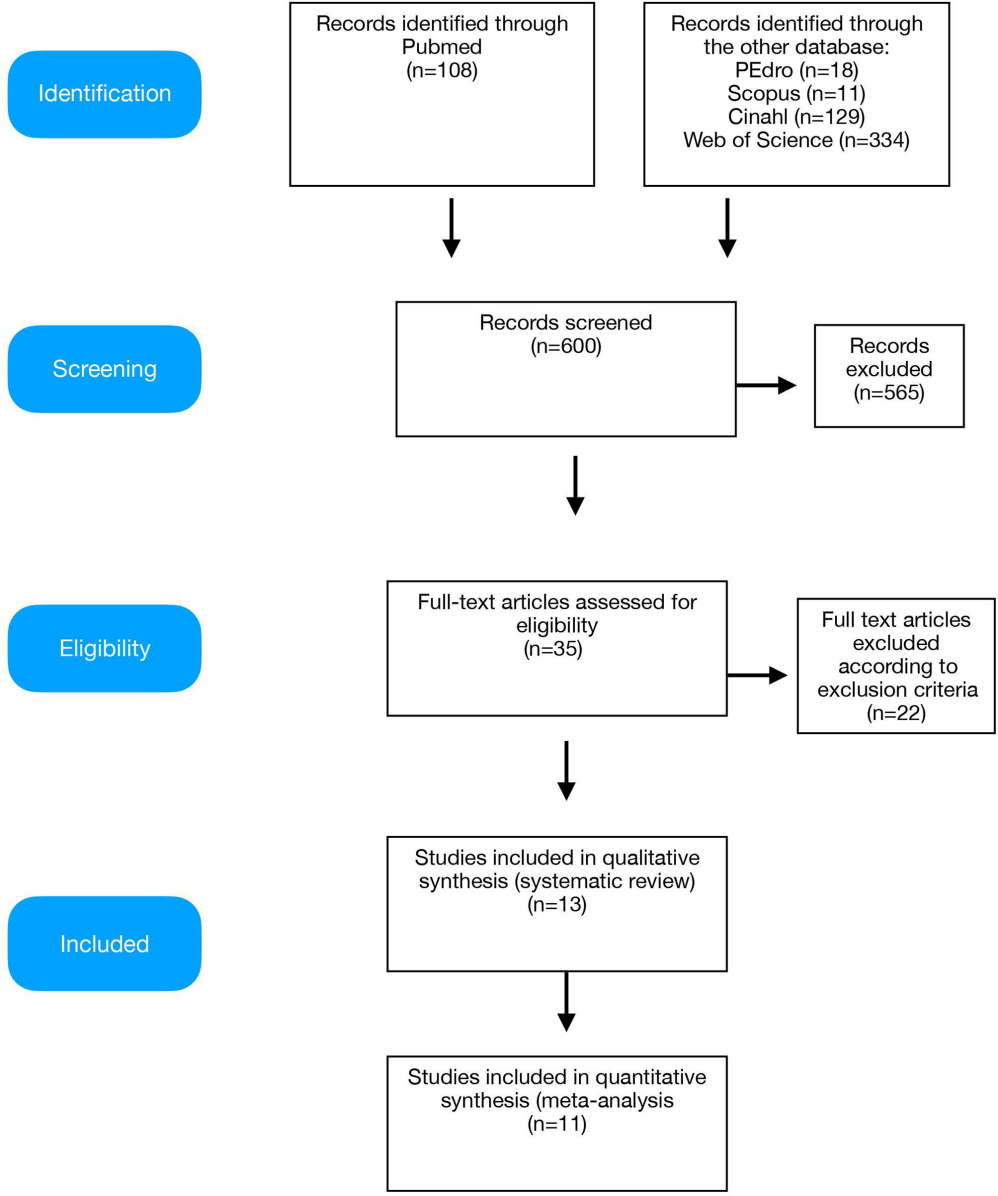
Preferred Reporting Items for Systematic reviews and Meta-Analysis (PRISMA) diagram of the study.

Database search identified 600 records; 565 papers were excluded because they were duplicates or not fulfilling the eligibility criteria. Among the remaining 35 papers that were obtained for full-text screening, 13 studies met the inclusion criteria and were included in the systematic review, while 11 studies were included in the quantitative synthesis (meta-analysis) (Figure 1).

### Methodological Quality of the Included Studies

The evaluation of each study, according to the PEDro criteria, is shown in Table 1. The methodological quality of the studies included in this review was globally more than acceptable (average total score 5.5 ± 1.0, range 4–7). Here, 46.2% of trial reports are moderate to high quality, scoring ≥6/10 on the PEDro scale (34, 37, 38, 40, 41, 43).

**Table 1:** Methodological quality of the included studies (PEDro criteria).

**References**	**Total score (0–10)**	**Methodological quality**	**Eligibility criteria**	**Random allocation**	**Concealed allocation**	**Baseline comparability**	**Blind subject**	**Blind therapist**	**Blind assessor**	**Adequate follow-up**	**Intention- to-treat analysis**	**Between-group comparisons**	**Point estimates and variability reported**
Mayr et al. (31)	5/10	Acceptable		X					X	X		X	X
Hornby et al. (32)	5/10	Acceptable	X	X	X	X						X	X
Hidler et al. (33)	5/10	Acceptable	X	X		X				X		X	X
Westlake and Patten (34)	6/10	Good	X	X	X	X				X		X	X
Uçar et al. (35)	4/10	Poor		X		X			X			X	
Van Nunen et al. (36)	4/10	Poor	X	X		X						X	X
Bang et al. (37)	7/10	Good	X	X	X	X			X	X		X	X
Bergmann et al. (38)	6/10	Good	X	X	X	X			X			X	X
Belas Dos Santos et al. (39)	5/10	Acceptable	X	X		X			X			X	X
Mayr et al. (40)	7/10	Good		X	X	X			X	X		X	X
Mustafaoglu et al. (41)	7/10	Good	X	X		X			X	X	X	X	X
Park et al. (42)	5/10	Acceptable	X	X		X			X	X		X	
Yun et al. (43)	6/10	Good	X	X	X	X				X		X	X

All participants were randomly assigned to the groups. In six studies (46.2%), the assignment of the subjects was carried out blindly (32, 34, 37, 38, 40, 43). In all papers (except for 55), the groups were similar at the beginning of the study regarding the most relevant prognostic indicators; of these, eight studies included blind assessors in evaluating at least one of the primary outcome measures of the study (31, 35, 37–42). In eight studies (61.5%), the results of at least one outcome measure were obtained in more than 85% of the subjects initially assigned to the groups (31, 33, 34, 37, 40–43). In all studies, the results of the intergroup comparison were reported for at least one of the main outcome measures. Mean and variability data for at least one of the main outcome measures were reported in 11 (84.6%) studies (31–34, 36–41, 43).

### Characteristics of Participants

The included studies involved a total of 445 subjects, of which 239 (53.7%) were randomly assigned to the experimental group treated with the Lokomat® and 206 (46.3%) were assigned to the control group, receiving conventional neuromotor rehabilitation and/or treadmill training. In the total sample, 171 (38.4%) were female and 274 (61.6%) were male; the mean age was 58.16 ± 2.38 years in the study group and 64.38 ± 2.19 years in the control group, without statistically significant differences.

Chronic stroke patients were enrolled in seven studies (53.8%) (32, 34, 35, 37, 39, 41, 42), while six papers (46.2%) involved subacute patients.

The characteristics of the participants of each study are shown in Table 2.

**Table 2:** Main features of the included studies.

**References**	**Study design**	**Participants**	**Rehabilitation treatment**	**Outcome measures**	**Results** **baseline – post-training**	**Follow-up**
Mayr et al. (31)	Randomized Study (crossover)	16 subacute stroke patients (10F/6M) *mean age 63.4 years*	SG (*n =* 8): 3 weeks Lokomat® + 3 weeks conventional therapy + 3 weeks Lokomat® CG (*n =* 8): 3 weeks conventional therapy + 3 weeks Lokomat® + 3 weeks conventional therapy *Lokomat*® *settings: BWS at 40%, speed 0.28 m/s, guidance force at 100%*. Treatments: 5 times/week	RMA Scale, “gross function”	RMA showed significantly more improvement in SG than in CG	None
Hornby et al. (32)	RCT	48 chronic stroke patients (18F/30M) *mean age 57 years*	SG (*n =* 24): Lokomat® CG (*n =* 24): treadmill with BWS assisted by therapist *Lokomat*® *settings: BWS at 40%, speed 2–3 km/h*. Treatments: 12 (30-min) sessions	Secondary outcome: BBS, mEFAP	CG facilitates greater improvements as compared to SG	6 months (results maintained)
Hidler et al. (33)	RCT	63 subacute stroke patients (24F/39M) *mean age 57.3 years*	SG (*n =* 33): Lokomat® CG (*n =* 30): Conventional treatment + treadmill *Lokomat*® *settings: BWS at 40%, speed 1.5 km/h, guidance force at 100%*. Treatments: 3 (1.5 h/session)/week for 8–10 weeks (total 24 sessions)	Secondary outcome: BBS, RMI	No significant differences between SG and CG	3 months (results maintained)
Westlake and Patten (34)	RCT	16 chronic stroke patients (2F/14M) *mean age 56.9 years*	SG (*n =* 8): Lokomat® CG (*n =* 8): treadmill [BWS 35%, speed 0.69 m/s (2.5 km/h)] *Lokomat*® *settings: BWS at 35%, speed 3 km/h, 100% guidance force*. Treatments: 3 (30 min/session)/week for 4 weeks	Secondary outcome: BBS, SPPB	SG significantly improved (*p* = 0.04) on the SPPB than CG; both groups significantly improved at BBS	None
U**ç**ar et al. (35)	Randomized study (parallel-group)	22 chronic stroke patients (22 M) *mean age 58.9 years*	SG (*n =* 11): Lokomat® CG (*n =* 11): conventional treatment *Lokomat*® *settings: BWS 50%, speed 1.5 km/h*. Treatments: 5 (30 min/session/week) for 2 weeks	TUG	SG significantly improved (*p* = 0.003) on the TUG than CG	6 weeks (results maintained)
Van Nunen et al. (36)	RCT	30 subacute stroke patients (15F/15M) *mean age 53 years*	SG (*n =* 16): Lokomat® (2 h/week; speed 2.5 km/h) + conventional therapy (1.5 h/week) CG (*n =* 14): conventional therapy according to guidelines (3.5 h/week) *Lokomat*® *settings: 1.5 km/h (up to 2.5 km/h), gradually decreased BWS (up to 10%) and decreased GF (up to 20%)* Treatments: 8 weeks	Secondary outcome: BBS, RMI, TUG	No significant differences between SG and CG	24 and 36 weeks (results maintained)
Bang et al. (37)	RCT	18 chronic stroke patients (9F/9M) *mean age 53.6 ± 3.9 years*	SG (*n =* 9): Lokomat® + conventional therapy CG (*n =* 9): treadmill training without body support + conventional therapy *Lokomat*® *settings: BWS at 40%, speed 0.45 m/s*. Treatments: 5 (1 h/session/week) for 4 weeks	BBS, ABC scale	The BBS score (*p* = 0.048), and the ABC score (*p* = 0.017) were significantly higher in the RAGT group	None
Bergmann et al. (38)	RCT	30 subacute stroke patients (13F/17M) *mean age 71 years*	SG (*n =* 15): Lokomat® CG (*n =* 15): conventional treatment *Lokomat*® *settings: BWS 50%, speed 2 km/h, guidance force at 100%*. Treatments: 5 (1 h/session/week) for 2 weeks	Secondary outcome: POMA-B	SG demonstrated significantly greater improvement (*p* < 0.05) than CG	2 weeks (results maintained)
Belas Dos Santos et al. (39)	RCT	15 chronic stroke patients (4F/11M) *mean age 50.8 ± 13.3 years*	SG (*n =* 7): Lokomat® + conventional therapy GC (*n =* 8): walking training by the therapist + conventional treatment *Lokomat*® *settings: BWS 50%, speed 1.5 km/h*. Treatments: 3 [1 h/session (2/h conventional treatment, 1/h walking training)] per week for 5 months	BBS, TUG	No significant differences between SG and CG	None
Mayr et al. (40)	RCT	66 subacute stroke patients (31F/35M) *mean age 68 years*	SG (*n =* 36): Lokomat® + conventional treatment CG (*n =* 30): conventional treatment + walking training *Lokomat*® *settings: BWS 40%, speed 1.2/2.6 km/h, guidance force at 100%*. Treatments: 5 (2 h/session/week) for 8 weeks	Primary outcome: mEFAP Secondary outcome: RMI, MM	No significant differences between SG and CG	None
Mustafaoglu et al. (41)	RCT	45 chronic stroke patients (13F/32M) *mean age 53.1 ± 13.2 years*	G1 (*n =* 15): Lokomat® 2 times/week + conventional treatment 5 days/week (45 min/session) G2 (*n =* 15): Lokomat® 2 times/week (45 min/session) G3 (*n =* 15): conventional treatment 5 days/week (45 min/session). *Lokomat*® *settings: BWS 40%, speed 1.2–2.6 km/h*. Treatments: 6 weeks	Primary outcome: BBS, TUG Secondary outcome: RMI	All primary and secondary outcome measures improved significantly in favor of G1, compared to G2 and G3 (*p* < 0.016)	None
Park et al. (42)	RCT	40 chronic stroke patients (15F/25M) *mean age 56.6 years*	SG1 (*n =* 12): Lokomat® + virtual reality + conventional therapy SG2 (*n =* 12): Lokomat® + metronome + conventional therapy CG (*n =* 16): Walking training with treadmill + conventional therapy. *Lokomat*® *settings: BWS 30%, speed 1.5–2 km/h, guidance force at 100%*. Treatments: 3 (45 min/session/week) for 6 weeks	BBS, TUG	In both SG1 and SG2 BBS and TUG significantly improved than CG (*p* < 0.05)	None
Yun et al. (43)	RCT	36 subacute stroke patients (17F/19M) *mean age 63.9 ± 8.2 years*	SG (*n =* 18): Lokomat® + visual feedback (virtual reality) CG (*n =* 18): conventional therapy (Bobath method) *Lokomat*® *settings: BWS 50%, speed 1.1 km/h, guidance force at 100%*. Treatments: 5 (30 min/session/week) for 3 weeks	Secondary outcome: BBS, PASS	BBS and PASS significantly improved in SG (*p* < 0.001 and *p* = 0.014, respectively)	1 month (results maintained)

*F, female; M, male; RCT, randomized controlled study; SG, Study Group; CG, Control Group; BWS, body weight support; BBS, Berg Balance Scale; ABC scale, Activities-Specific Balance Confidence scale; TUG, Timed Up and Go; RMA, Rivermead Motor Assessment; RMI, Rivermead Mobility Index; mEFAP, Modified Emory Functional Ambulation Profile; MM, Mobility Milestones; PASS, Postural Assessment Scale for Stroke; POMA-B, Performance-Oriented Mobility Assessment; SPPB, The Short Physical Performance Battery*.

### Characteristics of the Interventions in the Study Groups (Lokomat® Assisted Gait Training)

In seven studies (53.8%), training with the Lokomat® was combined with conventional physiotherapy and compared to conventional therapy (neuromotor rehabilitation according to stroke guidelines) (35–37, 39–42), while in the remaining six studies, patients performed training with the Lokomat® only compared to conventional physiotherapy (32–35, 38, 43).

The setting of the Lokomat® parameters was very similar in the different studies: body weight support initially 40–50%, then decreased during treatment by 30–40% until no weight support; initial gait speed about 0.4/0.5 m/s, gradually increased up to 0.7/0.8 m/s; 100% guidance, progressively decreased to 0%.

In one study, training with Lokomat® was combined with an augmented reality program by means of a monitor placed in front of the patients that provided a feedback on their performance through an avatar (42). In four other studies (30.8%), patients performing RAGT were provided with a feedback by means of monitors or mirrors (32, 33, 35, 43) that showed their performance parameters.

On average, treatment lasted 5–6 weeks. The study by Belas Dos Santos et al. (39) reported the longest total duration of the treatment, which lasted 5 months. The duration of each individual session was on average 1 h/day, specifically, 15 min for Lokomat® parameters setting and 45 min for training. The frequency of the sessions was 3–5 times/week.

The main characteristics of all the interventions are summarized in Table 2.

### Characteristics of the Interventions in the Control Groups (Conventional Rehabilitation Treatment)

Conventional treatment was very similar in all studies included in this review and was performed according to the main methods used in neurorehabilitation: Kabath, Bobath, and Perfetti. Specifically, the following activities were reported: stretching of the paretic lower limb, trunk control exercises, load balancing, exercises for static and dynamic balance, walking training. In four (30.8%) studies, the treadmill was used for locomotor training (33, 34, 37, 42), with speed settings similar to Lokomat® parameters. The duration of the treatment was on average 1 h/day, 3–5 times/week, for a total average duration of 5–6 weeks.

In all studies, intensity and frequency of rehabilitation treatment were comparable between the study group and the control group.

### Effects on the Balance

The first study that supported the effectiveness of Lokomat® was performed in subacute stroke patients, demonstrating significantly more improvement than conventional physical therapy in gross coordination of walking (31). The superiority of Lokomat® was not confirmed 2 years later by the study of Hidler et al. (33) who did not find any difference in balance between study and control group; moreover, conventional gait training interventions were shown to be even more effective than Lokomat® for both walking speed and distance. Also Hornby et al. (32) reported greater improvements both in single-limb stance time on the impaired leg and in speed in chronic stroke survivors who received therapist-assisted training compared to Lokomat®. Specifically, larger speed improvements were reported in subjects with less severe gait deficits.

In the pilot study by Westlake and Patten (34) involving chronic stroke patients, even if statistically significant differences were not apparent between Lokomat® and conventional therapy, significantly greater training-related improvements were reported in the Lokomat® group when considering balance as well as gait symmetry, walking speed, and lower extremity motor impairment and function. Some years later, another study suggested that patients undergoing Lokomat® training demonstrated significantly greater improvement on both static and dynamic balance and overground walking speed than those undergoing conventional training (35).

Van Nunen et al. (36) showed that in non-ambulatory subacute stroke subjects, Lokomat® training was as effective as conventional therapy in recovering walking ability, considering walking speed, balance, and function, while higher gains for Lokomat® were reported in chronic stroke subjects in improving balance, activity-specific balance confidence, and spatiotemporal gait parameters (gait speed, cadence, and step length) (37).

Comparable results between Lokomat® and conventional therapy were described in other studies assessing balance, coordination, and functional independence in individuals with ataxia after stroke (39) or locomotion and mobility in subacute non-ambulatory stroke patients (40), respectively.

Other studies showed greater improvement with Lokomat® training both alone and combined with conventional therapy when considering balance, mobility, and fear of falling in ambulatory chronic post-stroke patients (41) as well as a greater persistent reduction of pusher behavior compared to non-robotic physiotherapy (38).

When combined with augmented reality, Lokomat® training induced higher gain in balance and gait abilities in chronic stroke patients when compared to conventional treatment (42). Finally, Lokomat® proved to be more effective than conventional physical therapy in improving lateropulsion and balance function in subacute stroke patients (43).

Data from all the studies are reported in Table 2.

### Meta-Analysis

Pooling of results within meta-analyses was possible for the (i) BBS (*n* = 9), (ii) TUG (*n* = 5), and (iii) RMI (*n* = 4).

The characteristics of the different groups considered in the meta-analysis are reported in Table 3.

**Table 3:** Study groups included in the meta-analysis.

**Group**	**References**	**Outcome measure**
1	Hornby et al. (32); Hidler et al. (33); Westlake and Patten (34); Van Nunen et al. (36); Bang et al. (37); Belas Dos Santos et al. (39); Mustafaoglu et al. (41); Park et al. (42); Yun et al. (43)	Berg Balance Scale
2	Uçar et al. (35); Van Nunen et al. (36); Belas Dos Santos et al. (39), Mustafaoglu et al. (41); Park et al. (42)	Timed Up and Go
3	Hidler et al. (33); Van Nunen et al. (36); Mayr et al. (40); Mustafaoglu et al. (41)	Rivermead Mobility Index

**Figure 2:**
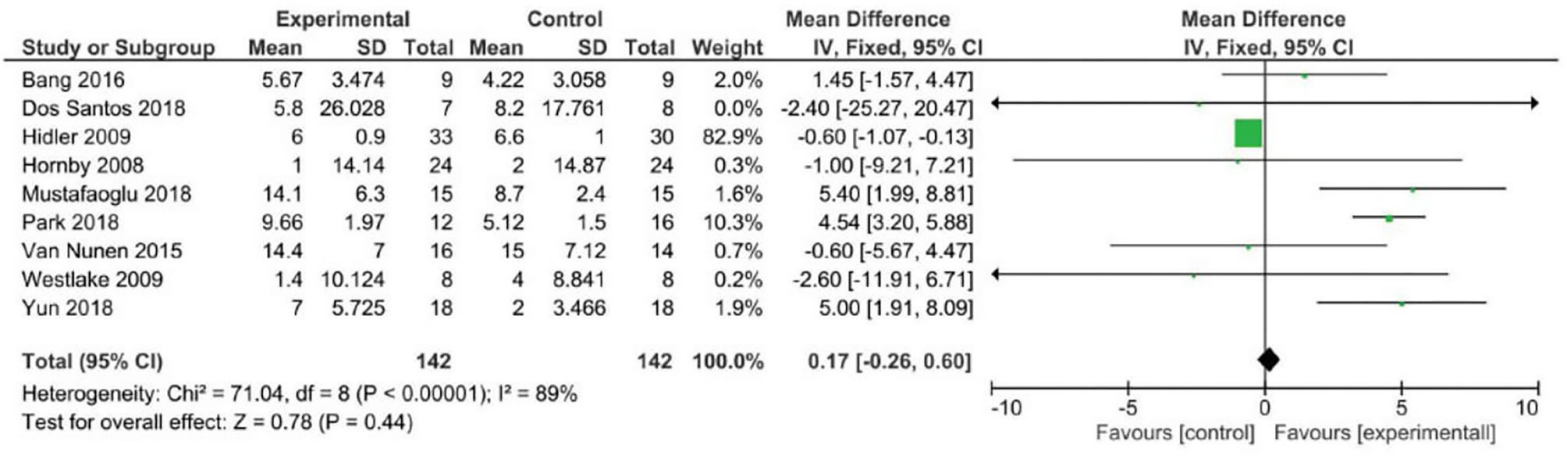
Forest plot for Berg Balance Scale (BBS).

**Figure 3:**
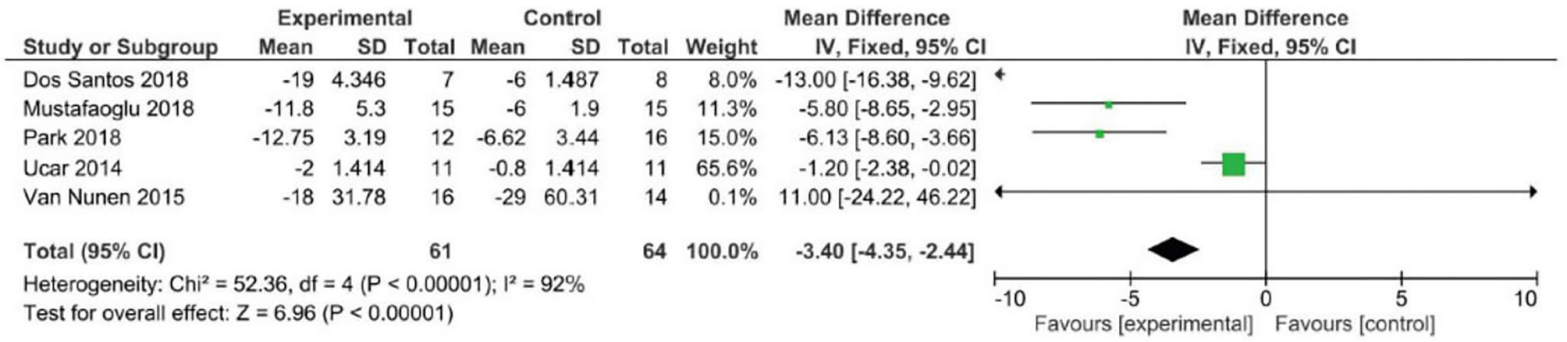
Forest plot for the Timed Up and Go (TUG) test.

**Figure 4:**
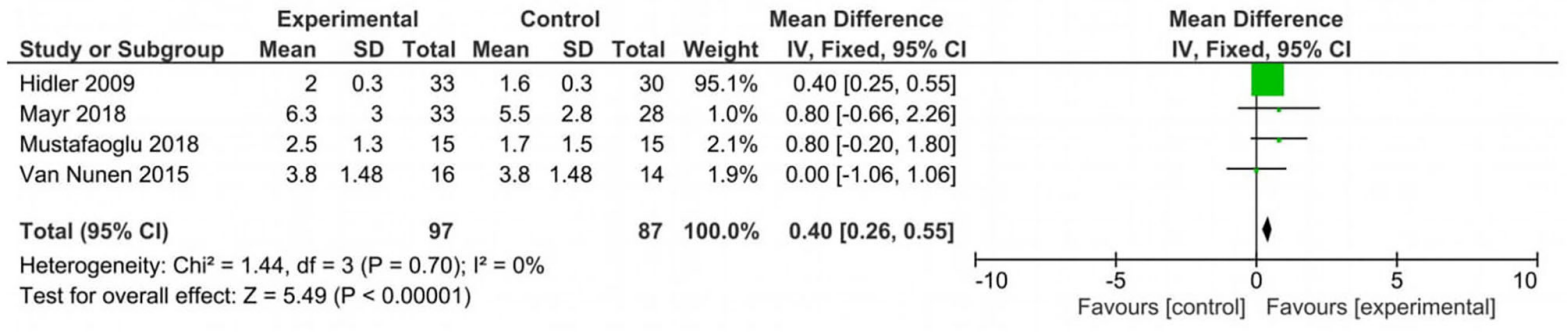
Forest plot for Rivermead Mobility Index (RMI).

Analyzing the effect of Lokomat® on the first outcome measure (BBS), the results of the meta-analysis showed no significant difference between study and control group and a high heterogeneity of the included studies (Figure 2). When considering mobility and static and dynamic balance measured by means of TUG, although the studies were heterogeneous, the results showed that the Lokomat® training determined a significantly higher improvement than that in the control group (pooled mean difference = −3.40, 95% CI −4.35 to −2.44; *p* < 0.00001; I2 = 92%, five trials, 61 participants) (Figure 3). Also, when evaluating the effect of the interventions on the RMI, the meta-analysis revealed a significant difference in favor of the study group (pooled mean difference = 0.40, 95% CI 0.26–0.55; *p* < 0.00001; I2 = 0%, four trials, 97 participants) (Figure 4).

## Discussion

The findings of this systematic review and meta-analysis showed that RAGT performed by means of Lokomat® might induce beneficial effects on balance in individuals affected by stroke similarly to conventional physical therapy. Moreover, data showed that the improvements occur in both acute and chronic stroke patients with different levels of impairment.

In general, as for other robotic exoskeletons, Lokomat® provides high-intensity and task-oriented exercise, allows to perform a gait cycle training also for not independent ambulators, and enhances motor re-education through a multisensory stimulation that involves patients in motivating activities (61, 62).

According to modern principles of gait rehabilitation, Lokomat® guides patients' legs through a preprogrammed physiological gait model that promotes the extension of the hip, encourages the start of the swinging phase, and promotes the step maximizing the load on the lower limbs. So the subjects experience an almost physiological proprioceptive input during walking (63, 64), transferring sensory–motor information to the central nervous system and inducing plastic changes (65–67). In fact, according to the basics of neuroplasticity and motor learning, robotic technologies, by providing controlled, repetitive, and task-specific stimulation, may induce adaptive modifications and reorganization of neural connections and networks, maximizing recovery and functional outcomes. Evidence suggests that robotic guided movements, in addition to typical motor areas, activate also deep neural centers such as the insula and the amygdala that are involved in movement memory and motivation to the movement and play a key role in inducing sensory processes related to the internal representation of the movements (68). Therefore, the simultaneous activation of sensory and motor systems could facilitate perceptual–motor skill learning and transfer (69).

As gait and balance are strictly related due to the nature of bipedal locomotion in which a majority of gait cycle is spent in single-limb support (70), it is not surprising that the clinical outcome of Lokomat®, in addition to gait parameters, also may involve balance. Indeed, the improvements in balance measures induced by Lokomat® suggest a transfer of skill from a task-specific locomotor training to non-walking functional balance parameters. The concept of “reverse transfer” was just proposed in relation to the idea that repeated practice of high-intensity walking training may improve non-walking tasks such as static balance and postural stability (71). The results of most of the papers included in this review support this view and hint that the dynamic, high-intensity, controlled, and repetitive training provided by Lokomat® may favor additional carryover to recovery parameters of postural and balance control beyond gait performance.

In the face of these favorable results, this review nevertheless highlights the presence of conflicting data: three studies in fact found that Lokomat® had the same effectiveness on balance as traditional physiotherapy (33, 36, 39), while one study has even indicated a smaller effect than conventional rehabilitation (32). Really, some doubts about the efficacy of RAGT had already been posed a few years ago, particularly regarding the scarce control of the patient over the initiation of each step, the lack of variability in visuospatial flow as opposed to overground walking, and the poor active contribution of the patient, crucial for activity-dependent learning (72–75).

It should also be emphasized that since the studies were few and often conducted on small populations, the results of this meta-analysis could have been polarized by the studies with wider sample sizes. That said, it is also true that the results of the larger studies also are usually the most reliable and generalizable, so in our opinion, the results of this review are completely reliable.

Furthermore, some general considerations must be made, first of all in relation to the device itself and to the setting parameters. The Lokomat® models have changed slightly over the years (e.g., Lokomat® models at the beginning maintained firm the pelvis, the successive models move also the pelvis of the patients), and in recent years, in some cases, further devices have been added to the robot (e.g., a monitor for augmented reality or visual feedback). Despite this, the basic structure and the preprogrammed walking pattern drove by the Lokomat® did not change significantly (gait cycle timing as stance vs. swing phase, inter-limb and inter-joint coordination, appropriate limb loading) and therefore, in our opinion, also the conditions of use and effects are yet comparable among different models.

One of the main advantages of robots consists of the possibility of freely setting the parameters according to each patient's characteristics; however, this implies a great heterogeneity in training protocols among clinical trials, as emerged also in this review, with consequent difficulties in comparing them. In principle, therapists seem to progressively adjust the level of motor assistance, resistance, and guidance as the patient's condition modifies, always providing the minimum amount of motor power required and maximizing patients' strength, motor function, and endurance (76). Despite that the scientific community pointed out the need to standardize rehabilitation protocols in order to optimize robotic use, to date, clear guidelines to select parameters are lacking (61) also for Lokomat®, and the usual approach promotes the customization of treatment, adapting the robots to the patient's clinical condition.

When considering the outcome measures, the results of this meta-analysis revealed that the BBS did not capture any difference between the intervention and the control group, unlike the other scales (TUG and RMI).

With respect to this issue, BBS can have floor and ceiling effects in evaluating balance in post-stroke subjects, and other balance measures adjunct or in conjunction with BBS have been suggested in evaluating balance in post-stroke subjects (77).

As about half of the studies included in this review involved chronic stroke patients, the ceiling effect may have resulted in an underestimation of therapy effects in the higher functioning individuals, without intergroup differences. Unfortunately, the variety of outcome measures used in the literature restricted the number of studies that could be considered in the meta-analysis.

When to start the treatment after the onset of the disease and for how long represent other crucial issues.

Although previous studies suggesting the presence of a “critical window for recovery” within the first 3–6 months post-stroke (78) seemed to justify to stop the treatments in the chronic phase (79), data from studies included in this review did not support this view, showing that Lokomat® was effective in improving balance and gait abilities also in chronic post-stroke subjects (33–35, 37, 41), consistently with more recent evidence suggesting that the time window for recovery may extend beyond 1 year post-stroke (80).

In addition to the heterogeneity of the studies, this review showed that the lack of longitudinal evaluations for most of the papers represents a main limitation; however, the limited data of the six studies with a follow-up assessment (follow-up range 2–36 weeks) showed substantial maintenance of the results at the post-training evaluation (32, 33, 35, 36, 38, 43). As no study reported side effects attributable to the use of Lokomat®, it seems legitimate to argue that this robotic device has been shown to be safe and feasible for use with subacute and chronic stroke patients.

Overall, the results of this meta-analysis elicit some comments and criticisms useful to address future research.

While powered exoskeletons hold promise, the literature surrounding their use for balance recovery is only just beginning to gather, with the majority focusing on gait. Given that this is a relatively new intervention for stroke, the objective of this meta-analysis was to map the current literature surrounding the use of Lokomat® for balance rehabilitation in post-stroke individuals and to identify gaps in the research. As expected, there are only a small number of published studies relevant to this topic. Moreover, studies were quite heterogeneous, which makes interpretation of the results challenging. First of all, no data are available regarding the optimal Lokomat® protocol. Larger controlled studies are required to determine the optimal timing and protocol design that will maximize efficacy and long-term outcomes in stroke patients. In addition, type, side, and severity of stroke and comorbid conditions were not considered in this review because of the scarcity of studies in this area: RCTs in which training with acute, subacute, and chronic stroke patients with specific categories of disability is compared might provide more specific indications for the use of Lokomat®. Finally, it is not definitely clear whether Lokomat® leads to a better outcome in balance-related outcome measurements compared with other more conventional gait rehabilitation methods. Also, the effects on static and dynamic balance, the impact of such intervention on functional goals that are meaningful to the patient, the transfer of the functional gains to real-world settings, and the effect on health-related quality of life have not yet received a satisfactory answer.

In conclusion, according to the results from this study, due to the small number of high-quality studies, the limited samples, the lack of follow-up evaluations, and the inconsistent results, at the moment, there is insufficient evidence to conclusively advocate in favor or against use of Lokomat® to improve balance control in stroke patients. However, the findings of most studies suggest that Lokomat® increasing therapy dosage, intensity, number of repetition, execution of task-oriented exercises, and combining top-down and bottom-up approaches can represent a useful tool for the physiotherapist to promote plasticity and functional recovery. In this sense, the present review and meta-analysis may represent a starting point for the development of new well-based research, continuing to advance the clinical application of powered exoskeletons.

## Data Availability Statement

The original contributions presented in the study are included in the article/supplementary material, further inquiries can be directed to the corresponding author.

## Author Contributions

The study has been designed by FB, MB, and CZ. Data have been gathered by FB and MB. Data have been analyzed by FB and CZ. The manuscript has been drafted by MB and CZ. MS and DI revised the manuscript for important intellectual content. All authors approved the final version of the manuscript.

## Conflict of Interest

The authors declare that the research was conducted in the absence of any commercial or financial relationships that could be construed as a potential conflict of interest. The reviewer MI declared a shared affiliation, with no collaboration, with the author MS at the time of the review.
